# Supplementary figures and images for: Glucose deprivation reduces proliferation and motility, and enhances the anti-proliferative effects of paclitaxel and doxorubicin in breast cell lines in vitro
Source: PLoS One. 2022 Aug 2;17(8):e0272449. doi: 10.1371/journal.pone.0272449 (PMC9345370; doi:10.1371/journal.pone.0272449)

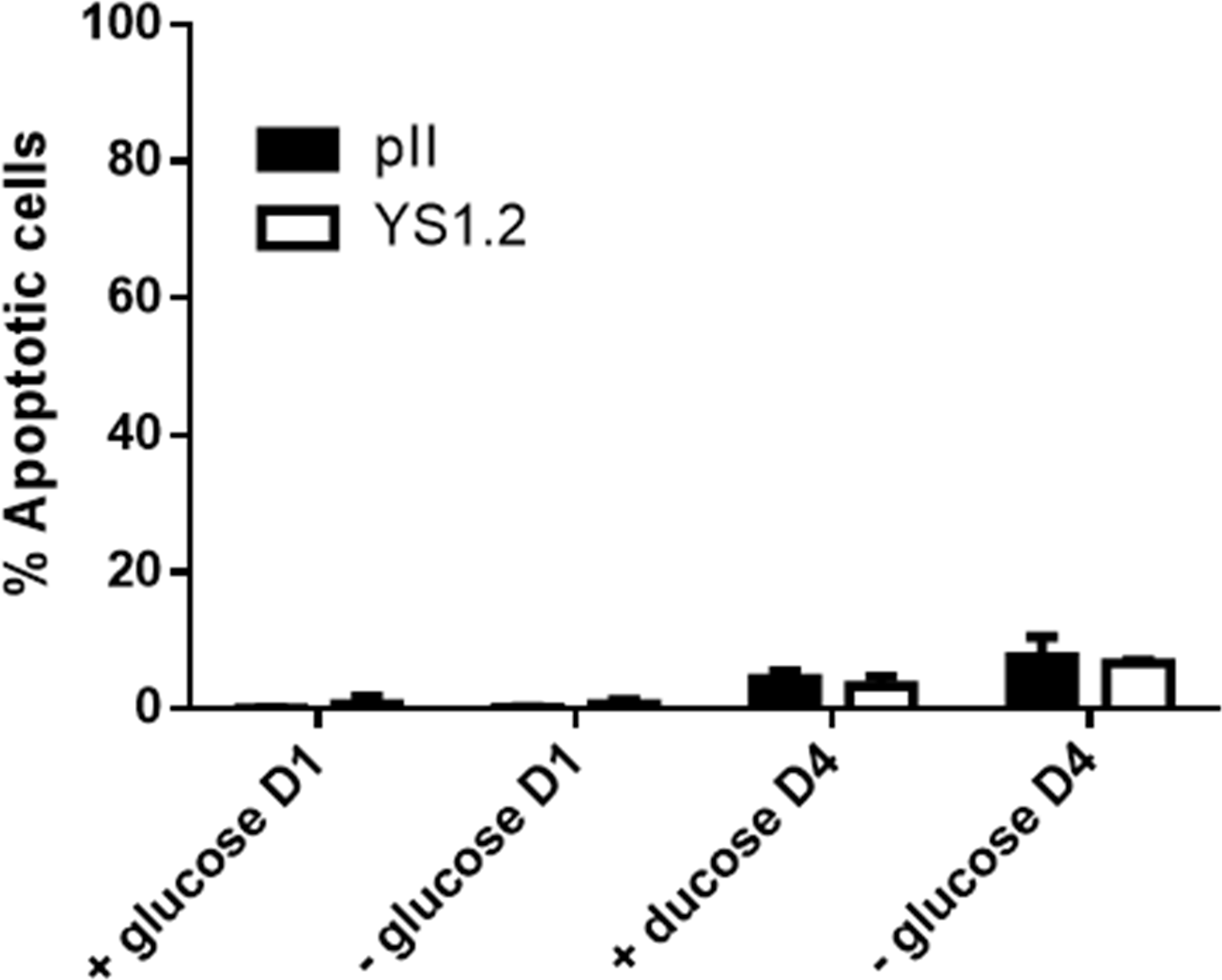

Supplement: S1 Fig — pII (solid bars) and YS1.2 (open bars) were cultured for 1 and 4 days in medium containing glucose (+ glucose) or without glucose (- glucose). Cell apoptosis was determined by flow cytometry using Annexin-V/7AAD staining as described in the methods. Histobars represent means ± SEM of at least 3 independent determinations. (TIF) [file pone.0272449.s001.tif]
