# Supplementary material for: Glucose deprivation reduces proliferation and motility, and enhances the anti-proliferative effects of paclitaxel and doxorubicin in breast cell lines in vitro
Source: PLoS One. 2022 Aug 2;17(8):e0272449. doi: 10.1371/journal.pone.0272449 (PMC9345370; doi:10.1371/journal.pone.0272449)
Supplement: Attachment — Submitted filename: Response to reviewer comments.docx [file pone.0272449.s003.docx]

Response to reviewer comments

Reviewer #1: The authors have adequately modified their manuscript. Besides, they also carefully answer the questions. Thereby, this paper could be acceptable for publication in this journal.

Response: we thank the reviewer for the positive comment.

Reviewer #2: The authors partially performed the suggested corrections, but the presentation of the cytotoxicity graphs compromises the veracity of the data due to the different optical densities existing between the cells. The initial difference between the control groups exceeds more than 40%, as suggested the data should be normalized. The different optical densities found in the graphs reflect the metabolic differences between tumor cell types. After normalization of optical densities, the values must be expressed in Percentage of Viability (%) in relation to the group of untreated (control cells).
I suggest using a program to normalize the data, for example the Graphic Pad Prism, or similar
Normalize the data to convert Y values from different data sets to a common scale. If you can't get Normalize to do what you want, take a look at the Remove Baseline analysis which can do some kinds of normalizing.
<https://www.graphpad.com/scientific-software/prism/>

Authors must correct the data presented in the way they are described, compromising their veracity

Response: We thank the reviewer for the valid comments. As per the recommendation we have modified the data presented in Fig 8 and 9 by considering the variability in the seeding density for vehicle treated cells with different glucose concentration in the culture medium. In the NEW modified Figs 8 and 9, vehicle treated cells cultured in glucose containing medium (25 mM, Black lines) is taken as 100% and the data are normalized accordingly.

The appropriate wording changes have been made in the ‘Results’ and ‘figure legends’ sections in the marked version of the manuscript.

We trust this clarifies the data and is what the Reviewer had in mind to be done.

Reviewer #3: The authors have responded to the addressed comments from my side and corrected the manuscript accordingly. No other comments

Response: we thank the reviewer for the positive comment.
