# Supplementary material for: Glucose deprivation reduces proliferation and motility, and enhances the anti-proliferative effects of paclitaxel and doxorubicin in breast cell lines in vitro
Source: PLoS One. 2022 Aug 2;17(8):e0272449. doi: 10.1371/journal.pone.0272449 (PMC9345370; doi:10.1371/journal.pone.0272449)
Supplement: Attachment — Submitted filename: Response to editor and reviewer comments 3rd round.docx [file pone.0272449.s004.docx]

**Response to editor and reviewer comments**

**Journal Requirements:**

Please review your reference list to ensure that it is complete and correct. If you have cited papers that have been retracted, please include the rationale for doing so in the manuscript text, or remove these references and replace them with relevant current references. Any changes to the reference list should be mentioned in the rebuttal letter that accompanies your revised manuscript. If you need to cite a retracted article, indicate the article’s retracted status in the References list and also include a citation and full reference for the retraction notice.

**Response: We do not understand the editorial remark concerning the references, as no issue was raised by any of the Reviewers in this context. Is this perhaps a new routine requirement by the journal to ensure that no retracted papers are cited?. We have double-checked all the references cited in this manuscript and to the best of our knowledge they are correctly cited and do not include any retracted papers that we are aware of. We actually checked each cited paper in PubMed and none of them were retracted.**

**Reviewer #2:**

**Old comments:**

The authors partially performed the suggested corrections, but the presentation of the cytotoxicity graphs compromises the veracity of the data due to the different optical densities existing between the cells. The initial difference between the control groups exceeds more than 40%, as suggested the data should be normalized. The different optical densities found in the graphs reflect the metabolic differences between tumor cell types. After normalization of optical densities, the values must be expressed in Percentage of Viability (%) in relation to the group of untreated (control cells).
I suggest using a program to normalize the data, for example the Graphic Pad Prism, or similar
Normalize the data to convert Y values from different data sets to a common scale. If you can't get Normalize to do what you want, take a look at the Remove Baseline analysis which can do some kinds of normalizing.

**New comments:**

To the authors of the manuscript, sorry for the insistence, the cell viability data must be corrected.
In the forwarded version, no changes were made to the figures. Therefore, I do not recommend publication, since the data presented were not analyzed and presented properly.

**Response:**

**We are very surprised by the reviewer’s comments regarding the cell viability data. In fact, we have carefully addressed *all* the comments previously raised by this and the other reviewers. We have responded to Reviewer two's comments regarding cell viability data presentation in Figures 8 and 9. There is some misunderstanding as we clearly submitted *corrected* figures as required by this reviewer. We are including in this letter the old and new versions of both figures to demonstrate the differences in the data presentation. We have considered the initial difference between the control (vehicle) groups cultured with different glucose concentration in the culture medium. In the NEW modified Figs 8 and 9, vehicle treated control cells cultured in glucose containing medium (25 mM, Black lines) is taken as 100% and the data are normalized accordingly to a common scale as the Reviewer suggested. This was our understanding of what the Reviewer had requested, and we have complied with it.**

**Although we do not perceive this to be the case, but if the continued objection is only the fact that the y axis is labelled as OD instead of cell number (?), we would point out that it is quite common practice for MTT data to be presented directly as OD values. We have published several manuscripts where we described MTT data in terms of OD readings (PMID: 34744727, PMID: 31980706, PMID: 30365135, PMID: 28276317, PMID: 26718772). Examples of recent papers where other authors have similarly presented MTT data as OD readings are PMID: 34951409, PMID: 34929154, PMID: 34916844.**

**But if there is something else that we have missed in his comment we would be grateful if the Reviewer could explain it more precisely and we would be happy to address the concern.**
